# Ethics review as a component of institutional approval for a multicentre continuous quality improvement project: the investigator's perspective

**DOI:** 10.1186/1472-6963-10-223

**Published:** 2010-07-30

**Authors:** Hanna Ezzat, Sue Ross, Peter von Dadelszen, Tara Morris, Robert Liston, Laura A Magee

**Affiliations:** 1 Department of Obstetrics and Gynaecology, University of British Columbia, Vancouver, V6 H 3N1, Canada; 2 Department of Obstetrics and Gynaecology, University of Calgary, Calgary, T2N 2T9, Canada; 3 Department of Family Medicine, University of Calgary, Calgary, T2N 4N1, Canada; 4 Department of Community Health Sciences, University of Calgary, Calgary, T2N 2T9, Canada; 5 Department of Surgery, University of Calgary, Calgary, T2N 2T9, Canada; 6 Department of Medicine, University of British Columbia, Vancouver, V5Z 1M9, Canada; 7 Department of Health Care and Epidemiology, University of British Columbia, Vancouver, V6T 1Z3, Canada; 8 Child and Family Research Institute of British Columbia, Children's & Women's Health Centre of BC, Vancouver, V5Z 4H4, Canada

## Abstract

**Background:**

For ethical approval of a multicentre study in Canada, investigators must apply separately to individual Research Ethics Boards (REBs). In principle, the protection of human research subjects is of utmost importance. However, in practice, the process of multicentre ethics review can be time consuming and costly, requiring duplication of effort for researchers and REBs. We used our experience with ethical review of The Canadian Perinatal Network (CPN), to gain insight into the Canadian system.

**Methods:**

The applications forms of 16 different REBs were abstracted for a list of standardized items. The application process across sites was compared. Correspondence between the REB and the investigators was documented in order to construct a timeline to approval, identify the specific issues raised by each board, and describe how they were resolved.

**Results:**

Each REB had a different application form. Most (n = 9) had a two or three step application process. Overall, it took a median of 31 days (range 2-174 days) to receive an initial response from the REB. Approval took a median of 42 days (range 4-443 days). Privacy and consent were the two major issues raised. Several additional minor or administrative issues were raised which delayed approval.

**Conclusions:**

For CPN, the Canadian REB process of ethical review proved challenging. REBs acted independently and without unified application forms or submission procedures. We call for a critical examination of the ethical, privacy and institutional review processes in Canada, to determine the best way to undertake multicentre review.

## Background

Ethics review of research involving human subjects is essential to protect the rights and safety of research subjects [1], by promoting socially beneficial research, protecting human subjects from harm and indignity, and maintaining trust between researchers and society [2]. In Canada, the Tri-Council Policy Statement (TCPS), "*Ethical Conduct for Research Involving Humans*" establishes a common policy of ethical conduct for research based on fundamental ethical principles [3]. Local research ethics boards (REBs) are responsible for reviewing research protocols at the institutional level, according to TCPS definitions of REB composition and function. This model is ideally suited to single centre research studies conducted by investigators based in that centre. However, multicentre collaborative research has become increasingly common [1,4,5], and local ethics review of multicentre studies has been criticised because it is time consuming and costly (for REBs and investigators), duplicates review effort, and has no discernible impact on improving patient safety [1,2,5,6].

Local review is further complicated by the need for review of institution-specific requirements, for example availability of institutional resources necessary for the research, and the requirements of provincial privacy legislation. In addition, some institutions require separate scientific review. The lack of standardized institutional review across Canada has complicated the review of multicentre research [6]. Many researchers have expressed frustration with the process and questioned its effectiveness [4]. Variability in ethics review has been documented in various settings [5], such as multicentre clinical trials [6-8], genetic epidemiology [9], and in both mailed and telephone survey research [10-13], but not in the context of database research where the primary concern is privacy, rather than specific issues of informed consent.

In 2005, the Canadian Institutes of Health Research (CIHR) funded the Canadian Perinatal Network (CPN) to develop a national database in Canada's 23 tertiary perinatal units. The objective of CPN is to improve quality of care by identifying best obstetric practices. The inaugural project examines threatened preterm birth. We evaluated CPN investigators' experiences with local institutional approval procedure to gain insight into the Canadian multicentre ethics review process for a database study, and to learn specifically how ethics review process is undertaken locally.

## Methods

CPN collects data, by chart review of maternal and fetal records, on all women who are admitted to a participating tertiary perinatal unit at 22^0^-28^6 ^weeks' gestation with threatened very preterm delivery. Personal identifiers are stripped during electronic data transfer to a central computerized database at the CPN Co-ordinating Centre, Vancouver.

From 2005-8, 16 tertiary care academic Canadian hospitals applied for REB approval for CPN. The protocol was unchanged over this time period. We obtained a complete file of REB applications and all correspondence between the CPN investigators, their local REB and the CPN Co-ordinating Centre. We did not contact REBs directly as we were not named on the relevant applications. LM (CPN co-principal investigator) and HE (Obstetrics and Gynaecology resident) independently constructed a timeline from submission to acceptance by the REB, catalogued the standardized content of each REB's application form and compiled a list of specific issues that the individual site investigator was asked to address by the REB. LM and HE were both at arms length from the REB process, other than the site-specific application at BC Women's Hospital (by LM): the new CPN Co-ordinator (TM) who was not involved in this application reviewed the BC Women's data for this analysis. Disagreement between reviewers was resolved by discussion and consensus.

We focused on issues of relevance to database research, determined *a priori *and identified in Sections 2 and 3 of the TCPS [3]: waiver of consent, accessing identifiable private information from hospital charts, appropriate safeguards for security and confidentiality (including data storage and destruction), confidentiality of reported data, and obtaining and linking data from several sources.

## Results

The number of steps involved in the initial application was not consistent across sites. Four sites had a one-step process involving only local REB application. Nine sites required an additional step of either local hospital or administrative approval. Three sites had a third step consisting of either separate scientific review before REB approval plus local hospital review, or REB, hospital plus other administrative or operational approval. In addition, six sites required sign-off by an additional department (information technology [IT] (n = 2), data access office (n = 1), privacy committee (n = 2), and/or communication department (n = 1). Five sites required separate health records approval. Two sites in different provinces involved a provincial privacy office but in both cases, those offices indicated that their involvement was unnecessary as patient protection fell to the hospital. At two sites, the IT department held up network installation of the database because of privacy concerns previously addressed by their local REB, resulting in delays of up to seven months.

Fifteen sites had an application form (all different) with specific questions asked. These questions are presented in Table 1. Three of 15 had specific forms for chart review or database studies; one site had a list of subheadings indicating the items to be addressed. Three of 15 sites asked about review by another REB. One site asked about previous peer review. All sites gathered basic information (e.g., investigator contact details). Fourteen sites asked about the sample size, method of patient selection, time period of the study, and funding source (which at one site was only whether the study was industry sponsored). Consent was a subheading or a question asked by all sites but with various levels of detail; six sites (40%) asked investigators to justify why the requirement for informed consent was waived if consent was not obtained (as for CPN).

**Table 1:** Standard elements covered on REB applications

Element	No. (%) of the 16 sites
**Basic Study Information**	
Purpose/objectives	9 (56%)
Signature of department head or supervisor	7 (44%)
Checklist of documents	7 (44%)
**Study Methods**	
Multicentre	10 (63%)
Study Design	9 (56%)
Location of research	12 (75%)
Description of procedures	9 (56%)
How outcomes measured	4 (25%)
**Subjects**	
Normal subjects enrolled?	4 (25%)
Inclusion/exclusion criteria	12/11 (75, 69%)
Risks	10 (63%)
Benefits	10 (63%)
Time required of subject	4 (25%)
Participant contacted by telephone or letter during study?	4 (25%)
**Privacy/Confidentiality**	
General description of privacy/confidentiality measures	6 (38%)
Conflict of interest	6 (38%)
Research staff credentials	6 (38%)
Independent peer review?	5 (31%)
**Budget**	
Fee for Service Payment details with sponsor	6 (38%)
Budget Details Requested	11 (68%)
Resource implications for hospital	4 (25%)

Table 2 shows that only some sites asked specific questions about data handling and privacy protection (although questions were later added to the form at one site). Six sites (40%) asked for a general description of privacy measures or for an assurance of confidentiality. There was no apparent relationship between the form version date and the detail requested.

**Table 2:** Specific questions relevant to databases

**Question**	**No. (%) of the 16 sites**
Identifying information used in stored data or in analysis?	4 (25%)
How is identity of subjects protected?	7 (44%)
Password protection for data?	2 (13%)
Is information going into a database?	5 (31%)
Are data linked?	5 (31%)
Does anyone outside site have access to data?	5 (31%)
Data monitoring procedures	5 (31%)
Who will analyze the data?	5 (31%)
Disposition of data at end of study	8 (50%)
Plans for future use of the data	5 (31%)
Privacy risks and controls assessment	3 (19%)

Overall, it took a median [range] of 42 days [4-443] to receive final REB approval. When CPN underwent expedited review (n = 9), the median time to approval was 33 days [8-239]. When local hospital approval was given separately from REB approval, the *additional *median time to approval was 32 days [4-197]. Ten REBs had no issues with the proposal: their median time to approval was 33 days [8-251]. For the site which required 251 days to receive approval despite no ethical concerns, REB approval took 54 days, but then local hospital approval took a further 197 days. Six REBs requested more information: their median time to approval was 178 days [55-443].

When more information was requested, one REB (of 10 that did not ask the question routinely) asked the investigators to justify why informed consent should be waived for CPN. Three sites had concerns about privacy beyond what was covered in the application: whether identifiers were recorded (n = 4), clarification regarding who had access to the data (n = 2), location of data storage (n = 1), future use of data (n = 2), and technical concerns about database operation (n = 1). Two of the sites were based within the same province: one REB approved the protocol, while the other said that CPN needed approval from the provincial privacy office until the previous REB's approval was brought to their attention. Other issues raised were: administrative issues including the need for additional signatures (n = 2); insufficient salary for the research assistant (n = 1); and grammatical, spelling, or layout issues with the patient information pamphlet (n = 2). One site raised a new issue each time the previous issue was addressed, requiring six different responses.

## Discussion

This paper describes our experience of seeking ethics approval for a Canadian multicentre minimum risk study (collecting anonymised data from hospital charts). We found a similarly "muddled Canadian landscape" to that described by Silversides for clinical trials [6]. In that report, the complexity found in review of invasive interventions includes lack of standardization of ethics review, lack of cooperation between REBs and differing privacy legislation [1,6]. In our study, we found that much duplication and additional work was required by sites and the CPN Co-ordinating Centre, causing significant delays before the CPN study was finally approved in all 16 participating sites, even though 10 REBs had only minor comments on the protocol.

The time, effort and cost of obtaining multiple ethics approvals have been highlighted by a number of authors. The resources consumed (by form filling and responding to questions from REB) add to the cost of research [4,14-16], and can delay both the initiation and completion of the research. The result of is an overall decrease in the amount of research undertaken, a cost borne by institutions and society more generally [14]. A high burden of the cost and effort is specifically borne by the REBs, including the paid REB staff and the volunteer members of the REBs who conduct the reviews and debate the ethics of studies [5,14].

Despite the cost of duplication of ethics review for researchers and REBs, there is no evidence that more review leads to either more ethical research, or greater patient safety [2]. Our study offers an insight to this argument: despite the delays caused as a result of institutional review, our study protocol was eventually adopted without substantive changes in all 16 sites. Indeed, our study highlights the types of queries that cause delays: REB procedural enquiries related to incomplete application forms, concern about the quality of the science, privacy issues and limited institutional resources. Unfortunately our study was a retrospective review of the ethics approval process, and we are unable to accurately determine the contribution that each aspect of review made towards the overall delays in receiving study approval.

Qualitative research involving interviews with investigators provides particular insight into the ethics review process, pointing out that ethics review can be divided into two integrated components: procedural issues, such as form design, checking for incomplete applications or consent forms; and ethics review proper [14,17]. It is important to remember that perceived flaws in the REB process cannot always be attributed to problems with the ethics review itself [15]. The procedural issues are generally what cause lack of trust and deterioration of relationships between researchers and REBs [14-18], and investigators may contribute significantly to delays in the review process. Many investigators consider ethics review itself to be positive, protecting the researchers as well as patients [2,15]. In fact, the ethical considerations seemed fairly similar between REBs in the CPN sites, underpinned by common ethical principles detailed in the TCPS in Canada [3]. In FDA inspections of REBs, the majority of citations are for procedural inconsistences, with few highlighting poor review of ethics [18]. It seems likely that differences between sites in procedural processes contribute to preventing collaboration in ethics review between sites [19].

Consensus exists that the ethics review should be proportionate, seeking to expend most effort on higher risk studies, with less deliberation devoted to studies considered to have no material ethical issues, or to involve minimum risk to patients [3,14,20]. Unfortunately the definition of lower risk is subjective, and it is this aspect of ethics review that might further hamper collaboration between REBs. In the case of CPN, collecting anonymised data from hospital charts in 16 sites, privacy was deemed the most significant concern, with REBs consistently raising issues related to privacy and informed consent. In particular, some REBs required assurance that the CPN Co-ordinating Centre would not have access to personal identifiers, although only five of the 15 standardized forms had questions specific to the handling of data and procedures for ensuring confidentiality. This could reflect differing roles of REBs in enforcing privacy legislation in different provinces and may indicate that privacy issues are evolving. Considerable controversy exists among REB chairs regarding access to medical records for research purposes [21].

The draft second edition of the TCPS recognizes the dangers caused by delays from multiple reviews of multicentre studies, and highlights the threats to implementing studies across institutions [22]. Several solutions have been suggested to reduce the inefficiency of local ethics review for multicentre studies. Centralised (national or regional) REBs have been proposed [17,23,24], offering the advantage of having a single administrative (and ethics) system for investigators to know and understand, and availability of specialised expert reviewers [4,17]. A combination of central review with local endorsement has also been suggested: this allows for local circumstances to be taken into account [23], but may lead to duplication of effort and causing delays in implementing research [24,25]. A novel suggestion by Nowak and colleagues, described a coordinated approach to ethics review, using a web-based program to promote communication between REBs which are part of a collaborative REB network [23]. Such a system might well reduce duplication of ethics review, but in order to succeed, a number of changes would need to be mandated. A national accreditation and regulatory system would be needed in any type of centralised or coordinated approach, ideally incorporating operational changes to ensure consistency between REB procedures [4,19,24,26,27]. To ensure consistency, national training would be needed for REB staff and committee members [4,19,24,27]. Unfortunately it will be difficult to set standards for ethical debate and to assess ethical decisions [26]. Only if REB accreditation was accepted, would REBs be able to trust each others' decisions, and accept approvals from other REBs [2,19]. The suggestions for centralised review will be unable to take account of local resource availability and privacy legislation that vary between provinces [6]. Québec has moved to a centralized provincial system, like the national system in the UK where local review boards handle only site-specific issues [28]. An alternative model, centralization by discipline, has been implemented by the Ontario Cancer Research Ethics Board; discipline-specific boards allow for expertise to develop in a specific area and for stakeholders to be involved in setting standards [29]. This model will disadvantage multidisciplinary research that involves different medical specialties.

Our results indicate that non-ethics review contributed significantly to the time taken to obtain ethics or institutional approval. In order to understand the contribution of local privacy and resource availability and review, detailed prospective studies are needed of the approval processes for multicentre studies. Investigators and REBs should be willing to contribute to such research that may lead to improvements in the review process.

Despite challenges in seeking ethics review, strategies exist to enhance collaboration between investigators and REBs, and enhance the review process [5]. Investigators should seek ethics training, particularly about the national ethics guidelines (TCPS in Canada) [3]. They should regard the REB members as advocates rather than adversaries, working with the REB, using their knowledge of the local REB system, privacy regulations, and other institution-specific requirements [5]. By working closely in collaboration with the REB to provide all the necessary information about their studies, the investigators will become local REB experts [5].

## Conclusion

Although the REB process varied between CPN sites, all REBs eventually approved the protocol without changes. In the context of publicly funded research, such as the CIHR-funded CPN, the additional delay and cost associated with obtaining multicentre REB approval may present an "unethical barrier" to potentially beneficial research and cause harm through the guise of "protection" by discouraging minimal risk activities that could improve care [4,30].

We believe that a critical examination of the ethical, privacy and institutional review processes in Canada are necessary, to explore their relative contribution to research delays. Only by divorcing ethics from the other types of more locally specific review could a centralised system of ethics review be workable in the Canadian context. CIHR and other funding agencies should exercise leadership to ensure that new Canadian models of multi-site ethics review are explored and implemented.

## Competing interests

The authors declare that they have no competing interests.

## Authors' contributions

All authors have read and approved the final manuscript. HE was involved in the study design, data analysis, interpreting the findings, and writing the manuscript. SR was involved in the study design, interpreting the findings, and writing the manuscript. PVD was involved in the study design, interpreting the findings, writing the manuscript. TM was involved in the study design and collection of data. RL was involved in the study design, interpreting the findings, and writing the manuscript. LAM was responsible for the initial conception and design of the study, interpreting the findings, and writing the manuscript.

## Pre-publication history

The pre-publication history for this paper can be accessed here:

http://www.biomedcentral.com/1472-6963/10/223/prepub
